# Cardiometabolic traits mediated the relationship from early life famine exposure to adulthood nonalcoholic fatty liver disease risk

**DOI:** 10.1017/S0007114521003342

**Published:** 2022-07-28

**Authors:** Xu Han, Jing Wang, Yaru Li, Dongsheng Hu, Meian He

**Affiliations:** 1 Department of Biostatistics and Epidemiology, School of Public Health, Shenzhen University Health Science Center, No. 1066 Xueyuan Avenue, Nanshan District, Shenzhen 518060, Guangdong, People’s Republic of China; 2 Department of Occupational and Environmental Health and Key Laboratory of Environmental and Health, Ministry of Education & Ministry of Environmental Protection, and State Key Laboratory of Environmental Health (Incubating), School of Public Health, Tongji Medical College, Huazhong University of Science and Technology, Wuhan 430030, People’s Republic of China

**Keywords:** Cardiometabolic traits, Famine, Mediation, Nonalcoholic fatty liver disease

## Abstract

Early life exposure to famine was associated with adulthood metabolic syndrome and nonalcoholic fatty liver disease (NAFLD), and NAFLD was also affected by cardiometabolic traits. However, the role of cardiometabolic traits in the associations from famine exposure to NAFLD was largely unknown. This study aimed to investigate whether the relationship between early life famine exposure and adulthood NAFLD risk was mediated by cardiometabolic traits. Overall, 7578 subjects aged 56·0 (sd 3·7) years in the Dongfeng–Tongji cohort were included and classified into late-exposed (1952–1954), middle-exposed (1954–1956), early-childhood-exposed (1956–1958), fetal-exposed (1959–1961) and non-exposed (1962–1966, reference) group according to the birth year. NAFLD was diagnosed by experienced physicians via abdominal B-type ultrasound inspection. Mediation analysis was used to evaluate the mediating effects of cardiometabolic traits. Compared with those non-exposed, after multivariable adjustment, participants in fetal-exposed group (OR: 1·37; 95 % CI 1·08, 1·73) had 37 % higher risk to develop NAFLD, and the overall childhood-exposed group had marginally significant association with NAFLD (OR: 1·39; 95 % CI 0·99, 1·94). Stratification analysis found the famine–NAFLD associations more evident in women and those born in areas severely affected by famine. Mediation analysis showed that cardiometabolic traits such as total cholesterol, TAG glucose index, *γ*-glutamyl transpeptidase, alkaline phosphatase and alanine aminotransferase mediated 6·7–22·2 % of the relation from famine exposure to higher NAFLD risk. Early life exposure to famine was related to increased adulthood NAFLD risk, and this relationship was partly mediated by cardiometabolic traits.

With rapid economic development and transitions of lifestyle, the prevalence of nonalcoholic fatty liver disease (NAFLD) is increasing dramatically in China^(1)^, with estimated cases from 246 million in 2016 to over 300 million cases in 2030^(2)^. NAFLD has progressively become one of the most common chronic liver diseases, leading to cirrhosis, liver cancer and even liver mortality^(3)^.

The Developmental Origins of Health and Disease postulates that adverse events occurring during early phases of human development increase disease risk through life, especially metabolic diseases^(4)^. China’s Great Famine may have been such an adverse event. The Great Famine, lasting for about 3 years (1959–1961), affected almost all people living in the Chinese mainland. It caused millions of excess deaths, and its detrimental effects persisted over a relatively prolonged period^(5,6)^. Previous studies have related famine exposure in early life to increased NAFLD risk in adulthood^(7–10)^. However, because of the limited numbers of studies, different grouping criteria for famine exposed people and different definitions of NAFLD, the relationship of early life exposure to famine and adulthood NAFLD needs further investigation.

The underlying mechanisms regarding how early life exposure to famine affects the development of NAFLD remain unknown. Accumulating evidence indicated that cardiometabolic traits (such as insulin, cholesterol and liver enzyme) are remarkable impact factors for NAFLD^(11)^. Several studies showed that early life exposure to famine was related to adulthood metabolic syndrome^(12,13)^. Therefore, it is possible that early life exposure to famine induces adulthood NAFLD risk via cardiometabolic traits. However, no study has been conducted to explore the potential role of cardiometabolic traits in the association between early life exposure to famine and adulthood NAFLD risk.

Therefore, to build upon preceding researches, we aimed to (a) evaluate the potential relationship of early life exposure to famine and NAFLD risk and (b) investigate the mediating effect of cardiometabolic traits on this potential relationship.

## Materials and methods

### Study population

Participants were selected from the Dongfeng–Tongji (DFTJ) cohort. The design, methods and other details of the DFTJ cohort have been described elsewhere^(14)^. Briefly, a total of 38 295 retirees were included and completed questionnaires, medical examinations and provided blood samples during the first follow-up from April to October 2013. Participants born after 1 October 1950 with specific birthdate were selected (*n* 12 538). Due to the ambiguous beginning and end of the Chinese famine, those born between 1 October 1958 and 30 September 1959 (*n* 781) and 1 October 1961 and 30 September 1962 (*n* 926) were omitted to minimise misclassification; those with cancer (*n* 534), chronic hepatitis (*n* 800), unclear B-type ultrasound inspection for NAFLD diagnosis (*n* 106) and drinkers with excess ethanol intake (≥ 140 g in men or 70 g in women per week in the past 12 months) (*n* 46) were further excluded. Eventually, 7578 individuals (1221 men and 6357 women with a mean age of 56·0 years) born between 1952 and 1966 remained for main analysis, and 9335 individuals (2438 men and 6897 women with a mean age of 58·3 years) remained for age-balanced analysis in the current study.

The present study was conducted in accordance with the Declaration of Helsinki. The study has been approved by the Ethics and Human Subject Committee of the School of Public Health, Tongji Medical College, Huazhong University of Science and Technology and Dongfeng General Hospital, the Dongfeng Motor Corporation. All study participants provided written informed consents.

### Data collection

In the DFTJ cohort, similar epidemiological questionnaires were used at baseline and follow-up to collect demographic characteristics, family and personal disease histories and lifestyle factors including smoking status, drinking status, physical activity, egg intake, red meat intake, vegetable intake and fruit intake. Physical activity levels were assessed as metabolic equivalent (MET) hours per week. MET were calculated by MET coefficients of activity × duration (hours per time) × frequency (times per week) and then converted to five grades according to quintiles. Individuals whose MET were at the upper two quintiles were defined as physical active.

Body weight, standing height and waist circumference were measured with participants in light indoor clothing and without shoes by trained staff using calibrated instruments (weight and height were measured using Dekon DK-08-E, Rayweigh; waist circumference was measured using Hoechstmass). Systolic blood pressure and diastolic blood pressure were measured with mercurial sphygmomanometer in one arm while the subject was seated after resting for about 10 min. Hepatitis B test including HBsAg, HBsAb, HBeAg, HBcAb and HBeAb was also performed at follow-up. Peripheral venous blood samples were collected after overnight fasting. Total bilirubin, total cholesterol (TC), TAG, HDL, LDL, *γ*-glutamyl transpeptidase (*γ*-GT), alkaline phosphatase (ALP), alanine aminotransferase (ALT) and aspartate aminotransferase were measured by the ARCHITECT ci8200 automatic analyzer (ABBOTT Laboratories. Abbott Park) using the Abbott Diagnostic reagents according to the instructions of the manufacturer. Fasting plasma glucose levels were measured with Aeroset automatic analyzer (by glucose oxidase method; Abbott Laboratories) at the hospital’s laboratory following standardised laboratory procedures.

### Exposure to famine and famine severity

Information about individual famine exposure was not collected in the DFTJ cohort; therefore, we classified famine exposure by birthdate and birthplace instead. During the 1950s and early 1980s, Chinese government rigidly controlled the population mobility, and only when individuals were employed or admitted to universities could migrate to other places^(15)^. The low population mobility made it feasible to examine the famine exposure by individuals’ birthdates and birthplaces. Consistent with previous Chinese famine studies^(12,16)^, participants were divided into five groups. Those born between 1 October 1962 and 30 September 1966 were classified as the non-exposed group; those born between 1 October 1959 and 30 September 1961 were classified as the fetal-exposed group; and those born between 1 October 1952 and 30 September 1958 (before the famine) were classified as late-, middle- and early-childhood-exposed groups by every 2 years.

The famine affected all provinces in the Chinese mainland, while famine severity was regionally different. On the basis of the mortality rates reported in preceding studies^(16,17)^, an excess mortality rate of 50 % was set as threshold value to differentiate the participants born in severely or less-severely affected area.

### Ascertainment of nonalcoholic fatty liver disease

All participants went through abdominal B-type ultrasound inspection (Aplio XG, Toshiba) by experienced physicians who had no knowledge of the study objective. To minimise the misclassification bias of alcoholic fatty liver, individual amount of alcohol consumption was calculated. The ethanol consumption in g per times was calculated as the sum of average ethanol content per type of alcoholic beverages multiplied by volumes of alcoholic beverages per times; the weekly average ethanol consumption was the product of ethanol amounts consumption per times multiplied by drinking frequency. The average ethanol content (v/v) of liquor, beer, wine and rice wine was 42, 4, 12 and 15 %, respectively^(18)^. NAFLD was diagnosed if the individuals met the ultrasound criteria for fatty liver, non-drinkers or drinkers with the ethanol intake less than 140 g in men (70 g in women) per week in the past 12 months^(19)^.

The severity of NAFLD was graded as mild, moderate or severe according to ultrasound criteria as follows^(20)^: mild NAFLD was defined as slight diffuse increase in fine echoes in the liver parenchyma with normal visualisation of the diaphragm and intrahepatic vessel borders. Moderate NAFLD was defined as moderate diffuse increase in fine echoes with slightly impaired visualisation of the intrahepatic vessels and diaphragm. Severe NAFLD was defined as marked increase in fine echoes with poor or no visualisation of the intrahepatic vessel borders, diaphragm and posterior portion of the right lobe of the liver. As the proportion of moderate (3·7 %) and severe NAFLD was small (1·5 %), we combined them into one group.

### Assessment of covariates

BMI was calculated as weight in kg divided by height in m^2^. To investigate the mediating effect of insulin, we calculated the TAG glucose (TyG) index instead, which has been suggested as a reliable surrogate marker of insulin resistance in previous studies^(21,22)^. The TyG index was determined as ln (TAG (mg/dl) × glucose (mg/dl)/2).

### Statistical analysis

In descriptive analyses, age was presented in means and standard deviations, MET were presented in medians (interquartile ranges) due to skewed distribution and categorical variables were presented in percentages. Differences in BMI, waist circumference, blood pressure, TC, TAG, fasting plasma glucose, TyG index, *γ*-GT, ALT, aspartate aminotransferase, ALP and total bilirubin between the exposed groups and non-exposed group were examined by generalised linear model and presented in adjusted least-square means and standard errors. The least-square means were compared by least significant difference.

Logistic regression model was used to calculate OR and 95 % CI of NAFLD. To reduce the bias related to age gaps between famine and post-famine births, an ‘age-balanced’ method was used, in which both pre-famine and post-famine births were included as non-exposed control subjects^(5,23)^. Stratification analyses were performed according to sex, BMI and famine severity. Interaction analyses between famine exposure and these covariates were also tested via adding a multiplicative factor in logistic regression models. Mediation analysis for cardiometabolic traits was performed via PROCESS procedure as described previously by Hayes^(24,25)^. A mediation analysis is to measure the direct effect of exposure and the indirect effect of exposure through mediator. The proportion mediated was calculated as the ratio of indirect effect to total effect.

Variables in multivariable adjusted model included sex (men or women), BMI (continuous), smoking status (never, former, current smoker), drinking status (never, former, current drinker), MET (continuous), famine severity (less severe, severe), egg intake (< 3 and ≥ 3 times/week), red meat intake (< 2 and ≥ 2 times/week), vegetable intake (< 14 and ≥ 14 times/week) and fruit intake (< 7 and ≥ 7 times/week). Data were analysed using SPSS 13·0 software (SPSS Inc.). *P* values and *P* for trend were calculated when famine groups entered analysis models as categorical or continuous variables separately; two-sided *P* value of < 0·05 was considered to be statistically significant.

## Results

### General characteristics of participants in descriptive analyses

Basic characteristics of participants according to famine exposure status are presented in Table 1. The overall prevalence of NAFLD was 37·8 % (2868 cases). Totally, 1164 (15·4 %) participants were exposed to the Great Famine in the fetal stage, and 5136 (67·8 %) were exposed during childhood. There were fewer men in the fetal-exposed and non-exposed groups. Compared with individuals non-exposed, those exposed were more likely to be smokers, drinkers and lowly educated (< 6 years); they also had higher levels of blood pressure, TC, fasting plasma glucose, TyG index, *γ*-GT, ALT, aspartate aminotransferase and ALP, as well as higher prevalence rates of NAFLD (both mild and moderate/severe).

**Table 1.** General characteristics of participants according to early life famine exposure status (*n* 7578) (Mean values and standard deviations)

Characteristics	Childhood-exposed groups						Fetal-exposed group		Non-exposed group	
	Late childhood		Middle childhood		Early childhood					
Birthdate	1952–1954		1954–1956		1956–1958		1959–1961		1962–1966	
*n*	1817		1570		1749		1164		1278	
Age, years										
Mean	60·2		58·1		56·2		53·1		49·7	
sd	0·6		0·6		0·5		0·6		1·3	
Women, %	73·4		79·6		84·7		96·0		91·9	
Education, %										
Primary school or below	18·6		12·3		7·8		2·6		1·9	
Junior high school	44·4		38·7		31·4		33·1		38·2	
Senior high school	26·7		36·9		49·3		57·9		51·4	
College or above	10·3		12·1		11·5		6·4		8·4	
Smoking status, %										
Never	81·6		85·0		87·4		96·0		93·0	
Former	5·0		3·3		2·6		0·5		0·8	
Current	13·5		11·7		10·1		3·5		6·2	
Drinking status, %										
Never	72·9		76·9		78·2		80·8		76·1	
Former	3·5		3·1		2·2		1·1		2·3	
Current	23·6		20·1		19·7		18·1		21·5	
Diet, %										
Egg intake (≥ 3 times/week)	66·7		69·4		66·7		67·7		67·4	
Red meat intake (≥ 2 times/week)	76·6		75·7		73·1		73·2		75·4	
Vegetable intake (≥ 14 times/week)	66·5		65·0		63·9		63·4		63·8	
Fruit intake (≥ 7 times/week)	58·6		62·1		63·0		66·8		66·7	
Metabolic equivalent, h/week										
Median	24		21		21		23		21	
IQR	31		33		32		32		34	

	Mean	sd	Mean	sd	Mean	sd	Mean	sd	Mean	sd
BMI, kg/m^2^ ^a^	24·5	0·1**	24·3	0·1*	24·3	0·1*	24·3	0·1*	24·0	0·1
WC, cm ^a^	84·4	0·4 × 8	84·3	0·2**	83·8	0·4**	82·6	0·5	82·4	0·4
SBP, mmHg ^b^	135·0	0·8**	131·9	0·8**	129·1	0·8**	127·6	0·9**	124·5	0·9
DBP, mmHg ^b^	80·6	0·5**	80·1	0·5**	79·1	0·5	79·2	0·5	78·7	0·5
TC, mmol/l ^b^	4·76	0·04**	4·79	0·04**	4·83	0·04**	4·72	0·05**	4·50	0·05
TAG, mmol/l ^b^	1·58	0·05	1·60	0·05*	1·63	0·05**	1·57	0·05	1·50	0·05
FPG, mmol/l ^b^	5·92	0·06**	6·04	0·06**	5·89	0·06**	5·86	0·07*	5·73	0·07
TyG index ^b^	8·72	0·02**	8·73	0·02**	8·73	0·02**	8·68	0·03**	8·59	0·03
*γ*-GT, units/l ^b^	31·4	1·3**	31·0	1·4*	31·2	1·4**	32·0	1·5**	27·9	1·5
ALT, units/l ^b^	22·1	0·6*	22·0	0·6*	22·4	0·6**	22·4	0·7**	20·8	0·6
AST, units/l ^b^	22·7	0·4**	23·1	0·4**	23·1	0·4**	22·6	0·5**	21·1	0·4
ALP, units/l ^b^	90·9	1·1**	89·2	1·1**	88·5	1·1**	85·5	1·2**	75·8	1·2
TBIL, umol/l ^b^	14·9	0·2	15·4	0·2	14·9	0·2	14·8	0·2	15·1	0·2
NAFLD, %	39·7*		39·7*		39·4*		37·8*		30·8	
Mild, %	32·1		32·2		31·1		28·1		24·2	
Moderate/severe, %	7·6		7·5		8·3		9·7		6·7	
Born in severely affected area, %	89·1		89·3		84·9		85·1		88·0	

WC, waist circumference; SBP, systolic blood pressure; DBP, diastolic blood pressure; TC, total cholesterol; FPG, fasting plasma glucose; *γ*-GT, *γ*-glutamyl transpeptidase; ALT, alanine aminotransferase; AST, aspartate aminotransferase; ALP, alkaline phosphatase; TBIL, total bilirubin.

a BMI and WC, least-square means and standard errors adjusted for sex, smoking status, drinking status, metabolic equivalent, egg intake, red meat intake, vegetable intake, and fruit intake.

b SBP, DBP, TC, TAG, FPG, TyG index, *γ*-GT, ALT, AST, ALP and TBIL, least-square means and standard errors adjusted for sex, BMI, smoking status, drinking status, metabolic equivalent, egg intake, red meat intake, vegetable intake and fruit intake.

Compared with the non-exposed group, *P < 0·05, **P < 0·01 (the least significant difference was used to adjust for multiple comparison).

### Association between early life exposure to famine and adulthood nonalcoholic fatty liver disease risk

As shown in Table 2, compared with those non-exposed, individuals in late- (OR: 1·48; 95 % CI 1·27, 1·72; *P* < 0·001), middle- (OR: 1·48; 95 % CI 1·27, 1·73; *P* < 0·001), early-childhood-exposed (OR: 1·46; 95 % CI 1·25, 1·70; *P* < 0·001) and fetal-exposed (OR: 1·36; 95 % CI 1·15, 1·61; *P* < 0·001) groups had higher risk of NAFLD after adjusting for sex (*P* for trend = 0·002). Additional adjustment for BMI and famine severity did not materially alter the results. In the fully adjusted model further adjusting for smoking status, drinking status, MET, egg intake, red meat intake, vegetable intake and fruit intake, individuals in late-, middle-, early-childhood-exposed and fetal-exposed groups had 39 % (OR: 1·39; 95 % CI 1·15, 1·66; *P* = 0·03), 52 % (OR: 1·52; 95 % CI 1·26, 1·83; *P* = 0·004), 45 % (OR: 1·45; 95 % CI 1·21, 1·74; *P* < 0·001) and 39 % (OR: 1·39; 95 % CI 1·14, 1·70; *P* = 0·004) higher risk of NAFLD, respectively (*P* for trend < 0·001). However, when age was introduced into the model, the above famine–NAFLD association altered to null. Meanwhile, we combined childhood-exposed groups into one group; the overall childhood-exposed participants had 45 % higher risk to develop NAFLD (OR: 1·45; 95 % CI 1·24, 1·70; *P* < 0·001). After further adjustment for age, the overall childhood-exposed group had marginally significant association with NAFLD (OR: 1·39; 95 % CI 0·99, 1·94; *P* = 0·05) while the fetal-exposed group still had 37 % elevated risk (OR: 1·37; 95 % CI 1·08, 1·73; *P* < 0·01).

**Table 2.** Associations of early life famine exposure with adulthood NAFLD risk (Odds ratio; 95 % confidence intervals)

	Childhood-exposed groups						Fetal-exposed group		*P* for trend	Childhood-exposed group		Fetal-exposed group		Non-exposed group
	Late childhood		Middle childhood		Early childhood									
	OR	95 % CI	OR	95 % CI	OR	95 % CI	OR	95 % CI		OR	95 % CI	OR	95 % CI	
Birthdate (main analysis)	1952–1954		1954–1956		1956–1958		1959–1961			1952–1958		1959–1961		1962–1966
Case/total, *n*	721/1817		624/1570		689/1749		440/1164			2034/5136		440/1164		394/1278
Model 1	1·48	1·27, 1·72**	1·48	1·27, 1·73**	1·46	1·25, 1·70**	1·36	1·15, 1·61**	0·002	1·47	1·29, 1·68**	1·36	1·15, 1·61**	1 (ref)
Model 2	1·36	1·14, 1·64**	1·51	1·25, 1·82**	1·45	1·21, 1·73**	1·38	1·13, 1·69**	< 0·001	1·44	1·13, 1·69**	1·38	1·13, 1·69**	1 (ref)
Model 3	1·39	1·15, 1·66*	1·52	1·26, 1·83**	1·45	1·21, 1·74**	1·39	1·14, 1·70**	< 0·001	1·45	1·24, 1·70**	1·39	1·14, 1·70**	1 (ref)
Model 4	0·65	0·29, 1·50	0·83	0·42, 1·64	0·91	0·54, 1·55	1·09	0·78, 1·51	0·003	1·39	0·99, 1·94	1·37	1·08, 1·73**	1 (ref)
Mild NAFLD														
Model 1	1·50	1·28, 1·76**	1·49	1·26, 1·76**	1·48	1·26, 1·73**	1·31	1·10, 1·57**	0·01	1·52	1·31, 1·75**	1·31	1·10, 1·57**	1 (ref)
Model 2	1·38	1·14, 1·66**	1·50	1·24, 1·82**	1·44	1·20, 1·74**	1·34	1·10, 1·65**	0·01	1·46	1·24, 1·72**	1·34	1·10, 1·65**	1 (ref)
Model 3	1·40	1·16, 1·68**	1·51	1·25, 1·83**	1·45	1·21, 1·75**	1·35	1·10, 1·66**	0·01	1·48	1·25, 1·74**	1·35	1·10, 1·66**	1 (ref)
Model 4	0·67	0·29, 1·54	0·84	0·42, 1·66	0·92	0·54, 1·57	1·06	0·76, 1·48	0·009	1·36	0·96, 1·92	1·31	1·03, 1·67*	1 (ref)
Moderate/severe NAFLD														
Model 1	1·34	0·95, 1·91	1·42	1·00, 2·03	1·36	0·96, 1·93	1·69	1·17, 2·43**	0·01	1·31	1·02, 1·68*	1·69	1·17, 2·43**	1 (ref)
Model 2	1·25	0·83, 1·87	1·57	1·05, 2·37*	1·48	0·99, 2·21	1·91	1·25, 2·92**	0·002	1·30	0·98, 1·74	1·91	1·25, 2·92**	1 (ref)
Model 3	1·28	0·85, 1·91	1·59	1·05, 2·39*	1·49	1·00, 2·23	1·93	1·26, 2·94**	0·002	1·32	0·99, 1·76	1·93	1·26, 2·94**	1 (ref)
Model 4	0·49	0·08, 3·01	0·74	0·17, 3·21	0·83	0·26, 2·62	1·42	0·71, 2·84	0·003	1·59	0·77, 3·28	2·00	1·22, 3·27**	1 (ref)
Age-balanced analysis	1952–1954		1954–1956		1956–1958		1959–1961			1952–1958		1959–1961		1950–1952/1966-
Model 1	1·13	1·01, 1·28*	1·13	1·00, 1·29	1·11	0·98, 1·26	1·03	0·89, 1·19	0·37	1·13	1·02, 1·24*	1·03	0·89, 1·19	1 (ref)
Model 2	1·14	0·99, 1·32	1·26	1·08, 1·47**	1·21	1·04, 1·40*	1·16	0·97, 1·38	0·01	1·20	1·07, 1·34**	1·16	0·97, 1·38	1 (ref)
Model 3	1·14	0·99, 1·32	1·25	1·07, 1·46**	1·20	1·03, 1·39*	1·15	0·97, 1·37	0·02	1·19	1·06, 1·33**	1·15	0·97, 1·37	1 (ref)
Model 4	1·14	0·99, 1·32	1·28	1·10, 1·50**	1·26	1·06, 1·49**	1·25	1·01, 1·55*	0·005	1·21	1·08, 1·37**	1·21	0·98, 1·49	1 (ref)

Model 1: Adjusted for sex.

Model 2: Additional adjustment for BMI and famine severity.

Model 3: Additional adjustment for smoking status, drinking status, metabolic equivalent, egg intake, red meat intake, vegetable intake, and fruit intake.

Model 4: Additional adjustment for age.

*P < 0·05; **P < 0·01.

In terms of NAFLD degrees, after multivariable adjustment, individuals in late-, middle-, early-childhood-exposed and fetal-exposed groups had 40 % (OR: 1·40; 95 % CI 1·16, 1·68; *P* = 0·003), 51 % (OR: 1·51; 95 % CI 1·25, 1·83; *P* < 0·001), 45 % (OR: 1·45; 95 % CI 1·21, 1·75; *P* = 0·002) and 35 % (OR: 1·35; 95 % CI 1·10, 1·66; *P* < 0·001) higher risk of mild NAFLD, respectively (*P* for trend = 0·01). The overall childhood-exposed participants had 48 % higher risk to develop mild NAFLD (OR: 1·48; 95 % CI 1·25, 1·74; *P* < 0·001). Only individuals in middle-childhood-exposed (OR: 1·59; 95 % CI 1·05, 2·39; *P* = 0·02) and fetal-exposed (OR: 1·93; 95 % CI 1·26, 2·94; *P* = 0·01) groups had elevate risk of moderate/severe NAFLD, which was probably due to the small sample size of moderate/severe NAFLD group. When age was further adjusted in model 4, the aforementioned famine–NAFLD association all changed to insignificant. However, when childhood-exposed groups were combined into one group and further adjusting for age, the fetal-exposed participants had 31 % (OR: 1·31; 95 % CI 1·03, 1·67; *P* = 0·03) and 100 % (OR: 2·00; 95 % CI 1·22, 3·27; *P* < 0·01) higher risk of mild and moderate/severe NAFLD.

### Age-balanced analysis

To reduce the bias related to age differences between famine and post-famine births, age-balanced analysis was conducted. On the one hand, we reclassified the non-exposed group including both pre-famine and post-famine births who were born between 1 October 1950 and 30 September 1952 as well as after 1 October 1966 (*n* 3449), and results remained statistically significant in middle- (OR: 1·25; 95 % CI 1·07, 1·46) and early-childhood-exposed (OR: 1·20; 95 % CI 1·03, 1·39) groups (*P* for trend = 0·02) (Table 2). When childhood-exposed groups were combined into one group, the overall childhood-exposed group had 19 % elevated risk of NAFLD (OR: 1·19; 95 % CI 1·06, 1·33). We additionally introduced age into the multivariable adjustment models. Significant associations were observed in middle- (OR: 1·28; 95 % CI 1·10, 1·50), early-childhood-exposed (OR: 1·26; 95 % CI 1·06, 1·49) and fetal-exposed group (OR: 1·25; 95 % CI 1·01, 1·55) (*P* for trend = 0·005). When childhood-exposed groups were combined into one group, significant associations were observed in the overall childhood-exposed group (OR: 1·21; 95 % CI 1·08, 1·37) while fetal-exposed group had marginally significant association with NAFLD (OR: 1·21; 95 % CI 0·98, 1·49). These results suggested that age might not be the only impact factor in the relationship of famine exposure and NAFLD risk.

### Stratification analysis

**Fig. 1.**
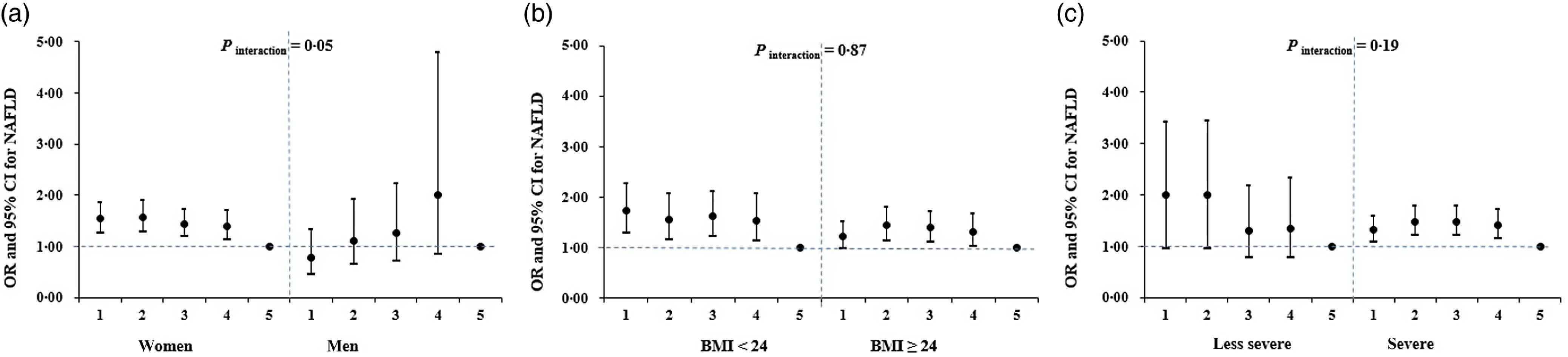
Associations between early life exposure to famine and adulthood NAFLD risk (ORs and 95 % CIs stratified by sex (a), BMI (b) and famine severity (c). Adjusted covariates included sex, BMI, smoking status, drinking status, metabolic equivalent and famine severity except for the stratified factors. 1, late-childhood-exposed group; 2, middle-childhood-exposed group; 3, late-childhood-exposed group; 4, fetal-exposed group; 5, non-exposed group.

We further conducted stratified analyses by sex, BMI and famine severity; results are presented in Fig. 1 and online Supplementary Table S1. After multivariable adjustment, the NAFLD risk increased 54 % (OR: 1·54; 95 % CI 1·26, 1·88), 56 % (OR: 1·56; 95 % CI 1·28, 1·91), 44 % (OR: 1·44; 95 % CI 1·19, 1·75) and 40 % (OR: 1·40; 95 % CI 1·13, 1·72) in late-, middle-, early-childhood-exposed and fetal-exposed women, respectively (*P* for trend = 0·01). The overall childhood-exposed women had 51 % elevated risk to develop NALFD (OR: 1·51; 95 % CI 1·28, 1·79). The famine–NAFLD associations were more evident in those born in regions severely affected by famine (late- (OR: 1·33; 95 % CI 1·09, 1·61), middle- (OR: 1·47; 95 % CI 1·21, 1·80), early-childhood-exposed (OR: 1·48; 95 % CI 1·22, 1·80) and fetal-exposed group (OR: 1·41; 95 % CI 1·14, 1·75); (*P* for trend < 0·001)). However, no significant interaction was found of famine exposure with any of the aforementioned factors on NAFLD risk.

### Associations of famine exposure, nonalcoholic fatty liver disease risk and cardiometabolic traits

The associations of early life exposure to famine with cardiometabolic traits based on general linear models are presented in online Supplementary Table S2. After multivariable adjustment, we observed that famine exposed groups had higher levels of TC, ALP (both *P* for trend < 0·001), TyG index (*P* for trend = 0·002), *γ*-GT (*P* for trend = 0·03) and ALT (*P* for trend = 0·02). We subsequently conducted logistic regression models to evaluate the relationship of cardiometabolic traits and NAFLD risk. As shown in online Supplementary Table S2, we found significantly positive associations between all these cardiometabolic traits and NAFLD risk except for total bilirubin (all *P* for trend < 0·05).

### Mediation analysis

As almost all the cardiometabolic traits were associated with NAFLD risk while only TC, TyG index, *γ*-GT, ALT and ALP were related with famine exposure, we conducted mediation analyses to assess whether the five contributors mediated the famine–NAFLD relation. As shown in Table 3, after controlling for potential confounders, TC, TyG index, *γ*-GT, ALT and ALP mediated the effect of early life famine exposure on the elevated adulthood NAFLD risk. The proportions mediated were 13·3, 22·2, 7·7, 6·7 and 7·4 %, respectively.

**Table 3.** Mediation analysis for cardiometabolic traits in the association from famine exposure to NAFLD risk (95 % confidence intervals; standard errors)

Mediators	Indirect effect	95 % CI	se	Direct effect	95 % CI	Percentage mediated
TC	0·009	0·005, 0·014*	0·002	0·057	0·014, 0·099*	13·3
TyG index	0·016	0·005, 0·026*	0·005	0·055	0·011, 0·100*	22·2
*γ*-GT	0·005	0·001, 0·011*	0·003	0·059	0·016, 0·102*	7·7
ALT	0·004	0·001, 0·009*	0·002	0·061	0·018, 0·104*	6·7
ALP	0·005	0·002, 0·009*	0·002	0·060	0·017, 0·103*	7·4

Results are obtained from mediation analysis after adjustment for age, sex, BMI, smoking status, drinking status, famine severity, metabolic equivalent, egg intake, red meat intake, vegetable intake and fruit intake.

* *P* < 0·05.

## Discussion

In this large population-based cohort of over 7000 Chinese adults, we found that early life exposure to the China’s Great Famine, especially during fetal stage and childhood, was associated with an increased risk of NAFLD in later life. Women who were exposed to famine in early life were more likely to develop NAFLD in adulthood than men. Assuming that the mediating effect holds, our estimates indicate that cardiometabolic traits (e.g. TC, TyG index, *γ*-GT, ALT and ALP) mediate 6·7–22·2 % of the associations between famine exposure and higher NAFLD risk.

Our results firmly supported previous findings that early life exposure to famine was significantly associated with increased risk of NAFLD^(7–10)^. However, the classification criteria for exposed and non-exposed groups in these studies were different. These studies simply classified famine exposed individuals into exposed^(7)^, or postnatal-exposed and prenatal-exposed groups^(10)^, or fetal-exposed and childhood-exposed groups^(8,9)^. In the present study, we specifically classified childhood-exposed people into early-, middle- and late-childhood-exposed groups by every 2 years and also conducted age-balanced analysis to reduce age differences. Furthermore, in terms of NAFLD diagnosis, we also calculated individual ethanol consumption and excluded those with excessive alcohol consumption to minimise misclassification bias. Although the associations of overall childhood-exposed group and NAFLD risk altered to marginal after adjustment for age, the OR of fetal-exposed group remained significant, confirming the deleterious effects of early life famine exposure on NAFLD.

In stratification and interaction analysis, the *P* value for interaction for sex was 0·051 (online Supplementary Table S1), suggesting that potential interaction might exist. We observed that the associations between early life famine exposure and increased NAFLD risk were women-specific, which was consistent with preceding studies^(8–10)^. Due to potential ability of oestrogen that increasing the cholesterol concentration^(26)^, women have higher possibility to develop hyperlipidaemia (78 % of patients with hyperlipidaemia in the present study were women). Another explanation might be mortality selection and son preference^(27,28)^. During the severe famine period, the mortality of men was higher than that of women, and the surviving men might have been healthier than those who prematurely died; moreover, in Chinese traditional culture, parents prefer to protect boys in tough circumferences, and girls might receive less food and care, which might result in worse health outcomes for women in adulthood^(27)^.

Systematic reviews^(5,29)^ have linked fetal famine exposure to the abnormalities of cardiometabolic conditions in adulthood. The ‘Fetal Origins hypothesis’ postulated that early adaptations in response to fetal malnutrition lead to metabolic and structural changes, which may increase the risk of chronic diseases in adulthood^(12)^. Additionally, it has been hypothesised that epigenetic dysregulation and inflammatory process related to prenatal malnutrition were associated with adverse metabolic phenotypes in later life^(30)^. Meanwhile, compelling and consistent evidence between cardiometabolic disturbances and NAFLD has been demonstrated in previous epidemiological studies^(31,32)^. Therefore, cardiometabolic traits may play a crucial role in the development of NAFLD caused by early life exposure to famine.

Some limitations to our study should be mentioned. First, participants were retired workers of the Dongfeng Motor Corporation, and the healthy worker effect might exist; conclusions might not be directly generalised to the general population. Second, detailed personal information about famine exposure, anthropometric measurements and lifestyle factors from birth to adulthood was not collected; thereby, the results provided evidence of association rather than causal relationship. Third, although the golden standard for the diagnosis of NAFLD is liver biopsy, it is impractical in such a large population. Ultrasonography is a feasible surrogate measure; meta-analysis showed that its sensitivity and specificity could reach 85 and 94 %^(33)^. In DFTJ cohort, NAFLD was diagnosed by ultrasound with experienced physicians, which minimised the misclassification of exposure. Fourth, despite results showed that childhood-exposed groups had higher NAFLD risk, it could not be concluded that fetal stage was not the critical and sensitive period. The China’s Great Famine lasted about 3 years; some participants in the fetal-exposed group also experienced the famine in infancy. Such misclassification might also attenuated the association between fetal exposure and NAFLD.

In conclusion, early life exposure to famine was related to higher adulthood NAFLD risk among Chinese population, and cardiometabolic traits mediated quite a few proportion of this relationship. Our study highlights a mediating role of cardiometabolic traits in such a relation between early life famine exposure and risk of adverse liver events. Future studies are encouraged to validate our findings and investigate potential mechanisms.

## Supplementary material

For supplementary material accompanying this paper visit https://doi.org/10.1017/S0007114521003342. click here to view supplementary material
