# Supplementary material for: Cardiometabolic traits mediated the relationship from early life famine exposure to adulthood nonalcoholic fatty liver disease risk
Source: Br J Nutr. 2021 Sep 1;128(2):244–51. doi: 10.1017/S0007114521003342 (PMC9301524; doi:10.1017/S0007114521003342)
Supplement: Supplementary file 1 [file S0007114521003342sup001.docx]

**Supplementary Table S1 Stratified Associations (ORs and 95% CIs) of Early Life Famine Exposure with NAFLD Risk**

|  | **Childhood exposed groups** | | | **Childhood exposed group** | **Fetal exposed group** | **Nonexposed**  **group** | ***P* for**  **trend** | ***P* for interaction** |
| --- | --- | --- | --- | --- | --- | --- | --- | --- |
|  | **Late childhood** | **Middle childhood** | **Early childhood** |  |  |  |  |  |
| Sex |  |  |  |  |  |  |  | 0.051 |
| Women | 1.54 (1.26-1.88)** | 1.56 (1.28-1.91)** | 1.44 (1.19-1.75)** | 1.51 (1.28, 1.79)** | 1.40 (1.13-1.72)** | 1 (ref) | 0.01 |  |
| Men | 0.79 (0.46-1.36) | 1.11 (0.64-1.94) | 1.27 (0.72-2.24) | 1.03 (0.31, 1.73) | 2.00 (0.84-4.81) | 1 (ref) | 0.01 |  |
| BMI |  |  |  |  |  |  |  | 0.87 |
| < 24.0 kg/m^2^ | 1.72 (1.29-2.28)** | 1.56 (1.16-2.08)** | 1.62 (1.22-2.14)** | 1.64 (1.28, 2.10)** | 1.53 (1.13-2.08)** | 1 (ref) | 0.03 |  |
| ≥ 24.0 kg/m^2^ | 1.22 (0.97-1.52) | 1.44 (1.14-1.82)** | 1.39 (1.10-1.74)** | 1.34 (1.10, 1.63)** | 1.31 (1.02-1.69)* | 1 (ref) | 0.01 |  |
| Famine severity |  |  |  |  |  |  |  | 0.19 |
| Less severe | 2.00 (0.96-3.45) | 2.00 (0.95-3.47) | 1.31 (0.79-2.19) | 1.65 (1.04, 2.61)* | 1.34 (0.77-2.34) | 1 (ref) | 0.98 |  |
| Severe | 1.33 (1.09-1.61)** | 1.47 (1.21-1.80)** | 1.48 (1.22-1.80)** | 1.43 (1.21, 1.69)** | 1.41 (1.14-1.75)** | 1 (ref) | <0.001 |  |

Results are adjusted for sex, BMI, smoking status, drinking status, famine severity, metabolic equivalent, egg intake, red meat intake, vegetable intake, and fruit intake except for the stratified factors;

**P* < 0.05; ***P* < 0.01

**Supplementary Table S2 Associations of Famine Exposure, NAFLD Risk with Cardiometabolic Traits**

| **Variable** | **Nonexposed** | | **Fetal exposed ^a^** | **Childhood exposed ^a^** | ***P* for trend** | **Without NAFLD** | **NAFLD ^b^** |
| --- | --- | --- | --- | --- | --- | --- | --- |
| BMI | 0 (ref) | 0.06 (-0.25, 0.37) | | -0.17 (-0.62, 0.28) | 0.52 | 1 (ref) | 1.59 (1.56, 1.62)** |
| WC | 0 (ref) | -0.49 (-1.18, 0.21) | | 0.87 (-0.15, 1.88) | 0.14 | 1 (ref) | 1.03 (1.03, 1.04)** |
| SBP | 0 (ref) | -1.26 (-3.18, 0.66) | | -3.45 (-6.25, -0.65) | 0.11 | 1 (ref) | 1.01 (1.01, 1.01)** |
| DBP | 0 (ref) | -0.68 (-1.79, 0.44) | | -1.77 (-3.40, -0.14)* | 0.25 | 1 (ref) | 1.02 (1.01, 1.02)** |
| TC | 0 (ref) | 0.24 (0.13, 0.34)** | | 0.33 (0.08, 0.18)** | <0.001 | 1 (ref) | 1.19 (1.14, 1.24)** |
| TG | 0 (ref) | 0.06 (-0.05, 0.18) | | 0.09 (-0.08, 0.26) | 0.10 | 1 (ref) | 1.52 (1.45, 1.59)** |
| FPG | 0 (ref) | 0.06 (-0.09, 0.21) | | 0.06 (-0.16, 0.27) | 0.24 | 1 (ref) | 1.19 (1.16, 1.23)** |
| TyG index | 0 (ref) | 0.06 (0.01, 0.12)* | | 0.08 (0.01, 0.16)* | 0.002 | 1 (ref) | 2.65 (2.45, 2.88)** |
| γ-GT | 0 (ref) | 3.79 (0.58, 7.01)* | | 3.04 (0.44, 7.73)* | 0.03 | 1 (ref) | 1.01 (1.01, 1.01)** |
| ALT | 0 (ref) | 1.30 (0.09, 2.62)* | | 0.85 (0.12, 2.92)* | 0.02 | 1 (ref) | 1.02 (1.01, 1.02)** |
| AST | 0 (ref) | 0.85 (-0.12, 1.83) | | 0.66 (-0.76, 2.08) | 0.05 | 1 (ref) | 1.00 (1.00, 1.01)* |
| ALP | 0 (ref) | 6.74 (4.12, 9.36)** | | 6.27 (2.45, 10.10)** | <0.001 | 1 (ref) | 1.00 (1.00, 1.00)** |
| TBIL | 0 (ref) | -0.27 (-0.78, 0.24) | | 0.07 (-0.68, 0.82) | 0.23 | 1 (ref) | 1.01 (1.00, 1.02) |

^a^ Results are derived from general linear models and presented as β with 95% CI adjusting for age, sex, BMI, smoking status, drinking status, famine severity, metabolic equivalent, egg intake, red meat intake, vegetable intake, and fruit intake except for the cardiometabolic trait itself.

^b^ Results are derived from Logistic regression models and presented as OR with 95% CI adjusting for age, sex, BMI, smoking status, drinking status, famine severity, metabolic equivalent, egg intake, red meat intake, vegetable intake, and fruit intake except for the cardiometabolic trait itself.

**P* < 0.05; ***P* < 0.01
